# Revelation of early detection of co-seismic ionospheric perturbations in GPS-TEC from realistic modelling approach: Case study

**DOI:** 10.1038/s41598-018-30476-9

**Published:** 2018-08-14

**Authors:** Dhanya Thomas, Mala S. Bagiya, Poikayil Sukumaran Sunil, Lucie Rolland, Anakuzhikkal Sudarsanan Sunil, T. Dylan Mikesell, Srinivas Nayak, Subrahmanyam Mangalampalli, Durbha Sai Ramesh

**Affiliations:** 1 Indian Institute of Geomagnetism, Navi Mumbai, India; 2 Université Côte d’Azur, OCA, CNRS, IRD, Géoazur, Sophia-Antipolis, Valbonne, France; 3 Environmental Seismology Laboratory, Department of Geosciences, Boise State University, Boise, Idaho USA; 4 Department of Geophysics, Andhra University, Visakhapatnam, India; 5 Department of Marine Geology and Geophysics, Cochin University of Science and Technology, Kochi, India

## Abstract

GPS-derived Total Electron Content (TEC) is an integrated quantity; hence it is difficult to relate the detection of ionospheric perturbations in TEC to a precise altitude. As TEC is weighted by the maximum ionospheric density, the corresponding altitude (hmF2) is, generally, assumed as the perturbation detection altitude. To investigate the validity of this assumption in detail, we conduct an accurate analysis of the GPS-TEC measured early ionospheric signatures related to the vertical surface displacement of the Mw 7.4 Sanriku-Oki earthquake (Sanriku-Oki Tohoku foreshock). Using 3D acoustic ray tracing model to describe the evolution of the propagating seismo-acoustic wave in space and time, we demonstrate how to infer the detection altitude of these early signatures in TEC. We determine that the signatures can be detected at altitudes up to ~130 km below the hmF2. This peculiar behaviour is attributed to the satellite line of sight (LOS) geometry and station location with respect to the source, which allows one to sound the co-seismic ionospheric signatures directly above the rupture area. We show that the early onset times correspond to crossing of the LOS with the acoustic wavefront at lower ionospheric altitudes. To support the proposed approach, we further reconstruct the seismo-acoustic induced ionospheric signatures for a moving satellite in the presence of a geomagnetic field. Both the 3D acoustic ray tracing model and the synthetic waveforms from the 3D coupled model substantiate the observed onset time of the ionospheric signatures. Moreover, our simple 3D acoustic ray tracing approach allows one to extend this analysis to azimuths different than that of the station-source line.

## Introduction

During an earthquake, the energy released by sudden vertical displacement disturbs the surrounding atmosphere. The resultant atmospheric disturbances that propagate upward are mainly acoustic and gravity waves. There are three main types of atmospheric wave perturbations which evolve during any earthquake/tsunami event: (1) a direct acoustic wave owing to a sudden vertical uplift or propagating rupture, (2) a secondary acoustic wave originating from propagating Rayleigh surface waves and (3) a gravity wave associated with the propagating tsunami^1–7^. The amplitudes of these seismic/tsunami induced atmospheric waves increase with altitude as they propagate upward in a region of decreasing atmospheric neutral density. Once the waves arrive at ionospheric altitudes, they redistribute the background electron density to generate perturbations known as co-seismic ionospheric perturbations (CIP)^4,5^.

The average arrival times of these individual atmospheric waves at ionospheric altitudes depend on the type of co-seismic wave. The upward propagation speed of acoustic waves depends mainly on the background atmospheric temperature along with the density and chemical composition of the atmosphere^8^. The acoustic waves induced either by the uplift around the epicentre or by the propagating Rayleigh surface waves require ≥10 minutes to arrive at the maximum ionospheric electron density altitudes (~280 km–~300 km), while gravity waves coupled to the propagating tsunamis typically require more than 30 minutes to an hour^2–4,6,7^.

The 11 March 2011 Mw 9.1 Tohoku earthquake (mainshock), one of the largest earthquakes recorded in modern seismology and geodesy, was preceded by the Sanriku-Oki earthquake (Sanriku-Oki Tohoku foreshock) of Mw 7.4, which occurred on 9 March 2011 at 02:45:20 UTC^9^. Interestingly, the Tohoku earthquake yielded CIP at times significantly less than 10 minutes (i.e. as early as ~420 s (~7 minutes)). These CIP were firstly attributed to fast propagating (supersonic) shock acoustic waves^10^. Subsequently, the scenario of CIP detection at lower altitudes using low satellite elevation geometry has been suggested^11^. However, no evidence/explanation of the responsible was given.

The Sanriku-Oki Tohoku foreshock event was recorded by a dense Global Positioning System (GPS) network and thus provides an opportunity to study the slip mechanism, surface deformation and its ionospheric manifestations^12,13^. We carry out a study of the ionospheric imprints of the associated surface deformation of this foreshock by exclusively focussing on early detection of foreshock CIP akin to the main shock. We demonstrate that the early detection of the foreshock CIP (<<10 minutes) at ionospheric altitudes is linked to the interaction of the seismo-acoustic wave with satellite line of sight (LOS) at altitudes lower than that of the maximum electron density (hmF2). Quantification of the observed early detection time is supported by a 3D acoustic ray tracing model involving the interaction between the upward propagating seismo-acoustic wave (in space and time) and satellite LOS. The significance of GPS station locations during the early CIP detection is analysed. To validate the proposed simple model, we reconstruct the CIP by integrating electron density perturbations modelled on a 3D spherical grid^14,15^ along the LOS, taking the ambient geomagnetic field and satellite motion into account. The synthetic slant TEC waveforms reproduce reasonably well the observed CIP onset and this suggests that the detected onsets are directly linked to the evolution of the acoustic wave in space and time.

### Characteristics of the Mw 7.4 Sanriku-Oki Tohoku foreshock

**Figure 1:**
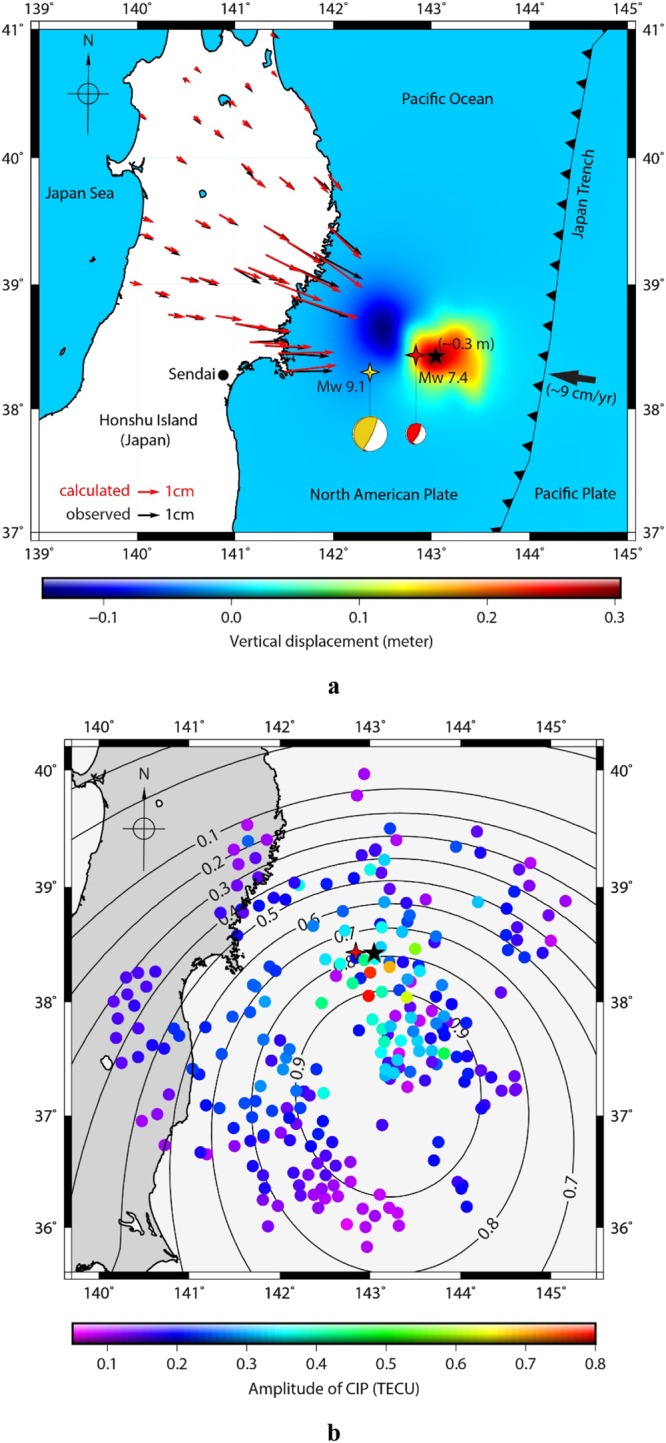
(**a**) GPS derived off-shore co-seismic vertical displacement near the epicentre (red four-point star) of the Mw 7.4 Sanriku-Oki Tohoku foreshock. Black five-point star indicates the location of maximum uplift modelled during the foreshock event, and the yellow four-point star represents the location of the Mw 9.1 Tohoku main shock. Black and red arrows represent the on-shore observed and modelled horizontal GPS velocity vectors, respectively. Yellow and red beach balls indicate the focal mechanism solutions of the main shock and foreshock, respectively. (**b**) CIP distribution around the maximum uplift during the Sanriku-Oki foreshock derived using the sTEC observations by GPS satellites PRNs (7, 8 and 10) from 173 GEONET stations (locations shown in Fig. S1). Black contour lines depict geomagnetic field-acoustic wave coupling factor derived at ~274 km altitude considering the maximum uplift area (black star) as the seismo-acoustic wave source. Coloured disks represent the peak-to-peak amplitude of the CIP. The Figure maps are generated using Generic Mapping Tools (GMT)^29^.

The Mw 7.4 Sanriku-Oki earthquake is the largest event of the foreshock sequence that preceded the Mw 9.1 Tohoku-Oki earthquake. It ruptured the subduction interface ~40 km northeast to the mainshock epicentre ~51 hours before^12^. During an earthquake, knowledge of the co-seismic surface deformation offers additional clues to unravel the corresponding manifestations in the ionosphere. Thus, we first evaluate the co-seismic ground deformation pattern during the Sanriku-Oki Tohoku foreshock. Figure 1a shows the foreshock epicentre (red four-point star) along with the epicentre of the Tohoku main event (yellow four-point star). The epicentres are after the USGS earthquake catalogue (https://earthquake.usgs.gov). The Japan Meteorological Agency (JMA) estimates the hypocentre of the foreshock at a depth of 8 km, and located about 25 km north and 36 km west of the Mw 9.1 Tohoku earthquake (see Fig. 1a). The rupture of this foreshock propagated west-northwest with an initial velocity of 3.1 km/s^12^. The co-seismic deformation pattern around the off-shore foreshock epicentral area is estimated using the method by Okada *et al*.^16^ and constrained using the observed GPS static displacements on land. The pattern shows uplift and subsidence east-southeast and west-northwest of the foreshock epicentre, respectively. The maximum deformation (~0.3 m uplift) is estimated ~20 km east of the epicenter, denoted by black star in Fig. 1a.

### Ionospheric manifestations of Mw 7.4 Sanriku-Oki Tohoku foreshock

Several wave types have been brought out in terms of lithospheric-atmospheric-ionospheric interactions using the dense GPS Earth Observation NETwork (GEONET), e.g. acoustic waves, acoustic-gravity waves, Rayleigh induced acoustic waves and tsunami induced gravity waves^2,6,17^. Further, information about the slip distribution from the ionospheric data, recorded by the same network, during the 11 March 2011 Tohoku earthquake has been obtained^10^. Our careful examination of slant TEC (sTEC) observations, from GEONET during the Mw 7.4 Sanriku-Oki Tohoku foreshock, reveals that multiple PRNs (7, 8 and 10) observed small, but detectable CIP during the foreshock as shown in Fig. 1b. The Figure shows the azimuthal distribution of the CIP’s first peak-to-peak amplitude at the ionospheric piercing points (IPP) around the epicentral area. In case of the Sanriku-Oki Tohoku foreshock, the IPP are considered to be located at ~274 km altitude based on the maximum electron density altitude (i.e., hmF2) derived from the International Reference Ionosphere (IRI)-2016 model^18^. It should be noted that CIP evolve preferentially south of the epicentre, i.e., in the region of favourable geomagnetic field-acoustic wave coupling as shown in Fig. 1b. The locations of GEONET GPS stations, which recorded these CIP, are presented in Fig. S1.

In general, azimuthal anisotropy of CIP amplitudes is well explained in the literature^3,4,14^. The non-tectonic forcing mechanisms, which mainly control the azimuthal anisotropy at ionospheric altitudes, are the geomagnetic coupling factor and the satellite LOS geometry. The geomagnetic field at ionospheric altitudes affects the coupling between the neutral atmospheric waves and ionospheric plasma through the Lorentz force. The Lorentz force acting upon the charged particle retards the movement of ionised particles perpendicular to the geomagnetic field at these altitudes. In the present case, a weak geomagnetic coupling factor is observed in the north (contours in Fig. 1b). As a result, the CIP amplitudes remain small in the north. On contrary, in the south, the strong coupling factor evidently leads to high amplitude CIP to the south. This is another example of equatorward propagation of CIP in addition to the two Nepal 2015 events previously reported^4,5^. Apart from the observed spatial asymmetry of CIP amplitudes (Fig. 1b), it can be noticed that these amplitudes remain smaller compared to the main shock and reach a maximum close to the uplifted area. This could be due to weak tectonic forcing owing to the associated small (~0.3 m) vertical crustal displacement induced by the Sanriku-Oki Tohoku foreshock. This is significantly smaller uplift compared to the Tohoku main shock, i.e., ~6 m^19^. The weak CIP in the far south of the foreshock maximum uplift are likely due to phase cancellation effects introduced by a non-favourable satellite geometry^4,14^. This aspect is not discussed further as the theme of the present paper is to discuss the early detection of CIP and not the amplitude variations.

**Figure 2:**
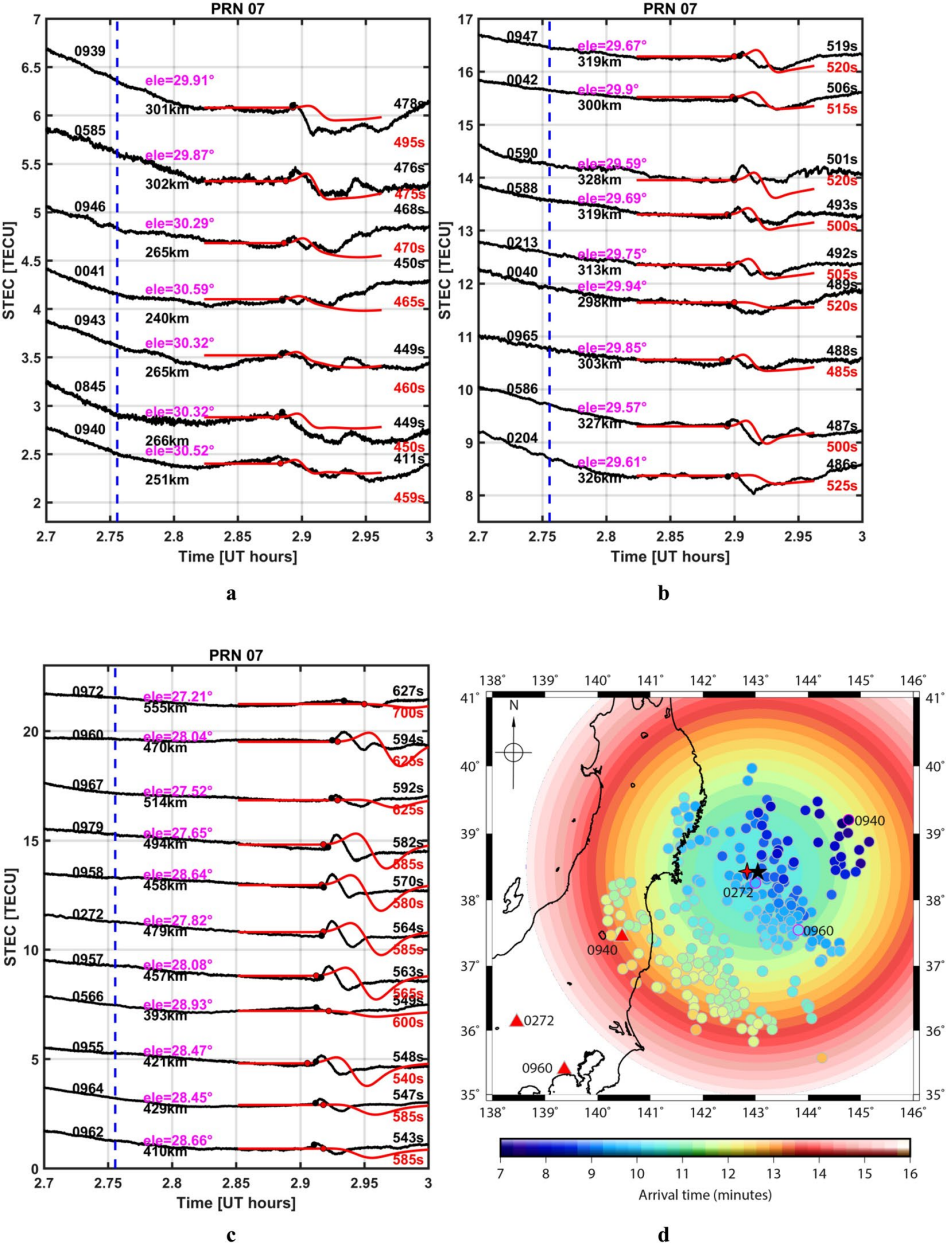
(**a**–**c**) sTEC time series as recorded by PRN 07 at various GEONET stations (black solid line). The sTEC exhibit significant CIP after the foreshock. The time series in red depict the synthetic CIP waveforms. The waveforms are generated over the 3D spherical grid, taking into account the moving satellite geometry and the ambient geomagnetic field. Each time series is labelled with respective GPS station number, station-source distance, the onset time of CIP in observations and in synthetic waveforms. The LOS elevation angle at the time of observed CIP onset is given in magenta. The dashed vertical blue line shows the onset time of the foreshock. (**d**) Comparison of the computed seismo-acoustic wave arrival time at an IPP altitude of ~274 km (coloured background) and the observed CIP onset time (coloured disks). The red four-point star denotes the foreshock epicentre, while the black five-point star indicates location of maximum uplift. The CIP onset times highlighted in magenta outline are studied in detail. The respective GPS observable stations for these highlighted CIP are presented with red triangles. The Figure map is generated using GMT^29^.

One second (1 s) slant TEC observations allow us to determine the onset time with better accuracy than the 30 s data. Therefore, we analyse the 1 s sTEC time series recorded by 27 GPS stations using satellite PRN 07 (Fig. 2a–c, black curves). We highlight that many of the CIP, depicted in Fig. 2a–c, were detected at times before 600 seconds after the foreshock onset. Figure 2a–c also contain the modelled CIP waveforms (red),constructed using the method proposed by Rolland *et al*.^14^ based on the acoustic ray tracing method of Dessa *et al*.^20^; which also takes the ambient geomagnetic field and geometry of PRN 07 into account. The CIP onset time in the observations and the synthetic waveforms are presented in Fig. 2a–c in black and red, respectively. It is interesting to see that the modelled time series replicate the observations fairly well both in arrival time and waveform shape.

### Approach to assess the observed early CIP detection

The focus of this study is to explain the early detection of CIP and identify the associated altitude through modelling. One of the major outcomes is that adopting a simple 3D acoustic ray tracing modelling approach, even more simple than the 3D coupled model used by Rolland *et al*.^14^, is sufficient to explain the early observed detection time feature. Firstly, we compute the space and time evolution of seismo-acoustic rays using a realistic velocity model and the Snell-Descartes law. Maximum crustal uplift (Fig. 1a) is considered as the source of the seismo-acoustic wave^21^. The vertical cross section of this evolution is shown in Fig. S2 along with the acoustic wave velocity profile used to derive the computed ray arrival times. These arrival times are henceforth referred as modelled arrival time (MAT). The MATs at an IPP altitude of ~274 km are presented in Fig. 2d as the coloured background. The black star in the Figure denotes the location of maximum uplift as the acoustic wave source. The observed detection time (ODT) for each CIP is represented by a coloured disk at the respective IPP location. In addition, we present the temporal evolution of CIP at every 25 s after ~400 s (~6.7 minutes) of the foreshock using Movie 1. From Fig. 2d, it is significant to note that ODTs of CIP toward north-northeast of the source deviate by more than ~100 s (~1.7 minutes) from the corresponding MAT, while they show reasonable correspondence in the south-southwest region.

**Figure 3:**
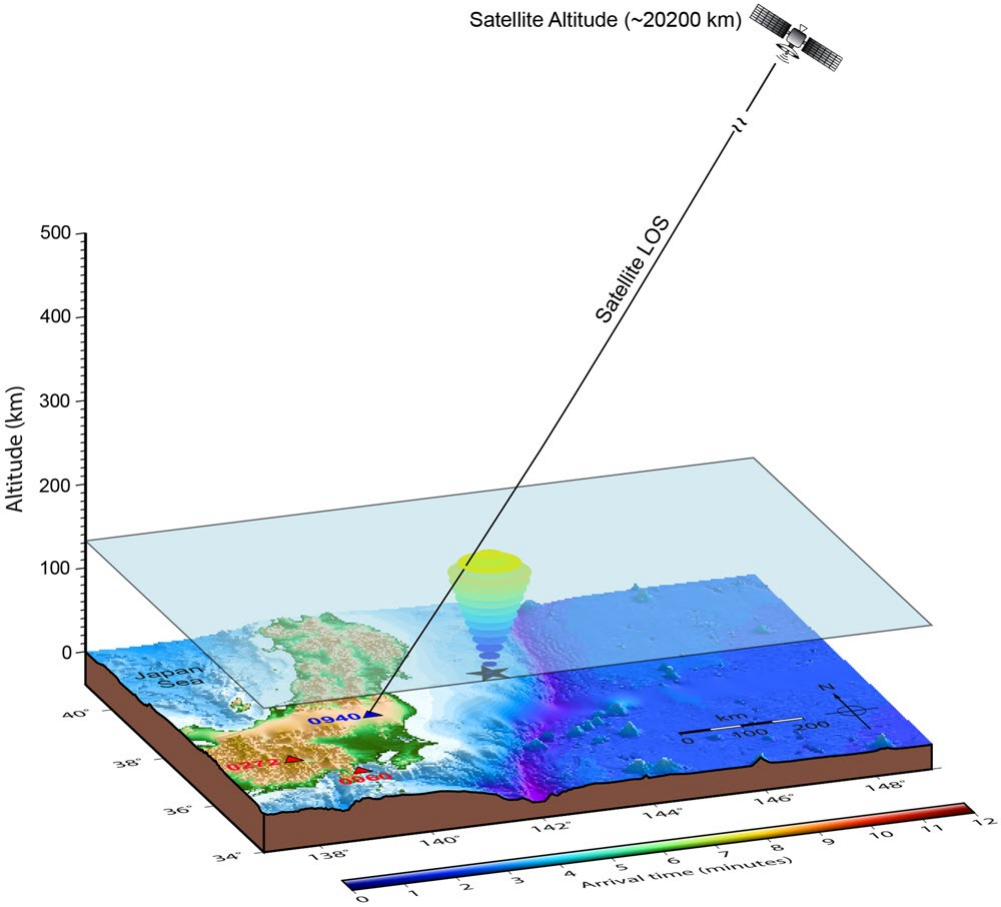
Proposed model containing the seismo-acoustic rays in 3D space and satellite LOS to explain the early CIP detection during the Sanriku-Oki Tohoku foreshock. The seismo-acoustic rays are computed in space and time by considering the maximum uplift as the source. The first interaction between the seismo-acoustic rays and PRN 07 LOS from GPS station 0940 is shown. PRN 07 LOS is plotted at the time of observed CIP onset. The altitude of the first interaction between the seismo-acoustic wave and the satellite LOS is highlighted with a transparent 3D plane. The maximum uplift location is denoted by the black star. The GPS station 0940 is located at a distance of ~251 km from this source. The elevation angle for PRN 07 at the time of CIP onset is ~30.5°. The conceptual orbiting plane of satellite PRN 07 is also shown. The base maps are produced using GMT^29^ based on 1 arc-minute ETOPO1 global relief model (www.ngdc.noaa.gov/mgg/global). We note that the separation between the station (0940)-source azimuthal plane and the station (0940)-satellite azimuth plane is ~2° at CIP onset at 0940 station.

In order to understand the cause of early detection of CIP, we explore the possibility of low elevation satellite geometry^11^ by proposing a 3D acoustic ray tracing model involving an interaction between the satellite LOS and the vertically propagating seismo-acoustic wave. In Fig. 3 we present all the components involved in our approach, such as the space and time evolution of seismo-acoustic ray traces in terms of MAT computed at every 1 km of atmospheric altitude and the satellite LOS. The satellite LOS is computed using the satellite navigation data and the receiver coordinates. The receiver coordinates are derived using the PPP mode. It is important to note that in Fig. 3 both horizontal and vertical axes tick contains the same unit of distance. The first interaction between the PRN 07 LOS from GPS station 0940 (highlighted in Fig. 2d) and the modelled ray arrivals could be noticed from the Figure. We compare the satellite LOS and the altitude of the ray arrival at each instant in time and determine the first interaction between the LOS and seismo-acoustic rays. We notice that the first interaction does not occur within ~411 s (~6.85 minutes), which is the observed CIP onset time in slant TEC data for this GPS station. Instead, we find that the first interaction between the LOS and the acoustic ray occurs at ~440 s (~7.33 minutes) at an altitude of ~131 km (Fig. 3). As the seismo-acoustic rays are presented in 3D space, we demarcate the first interaction using a transparent plane in the Figure for easier visualisation. It is pertinent to note that this arrival time is certainly early compared to the corresponding IPP altitude (~274 km) arrival time of ~720 s (~12 minutes) (Fig. 2d). Thus, we report that PRN 07 has started to detect the CIP at a lower altitude and an early time from station 0940 (~131 km, ~440 s) and not at the traditional IPP altitude (~274 km, ~720 s). It should be noted that observed onset time of the CIP at station 0940 and the time of an encounter between the PRN 07 LOS and the modelled acoustic ray differs by ~29 s. The probable cause of this residual difference is discussed later.

**Figure 4:**
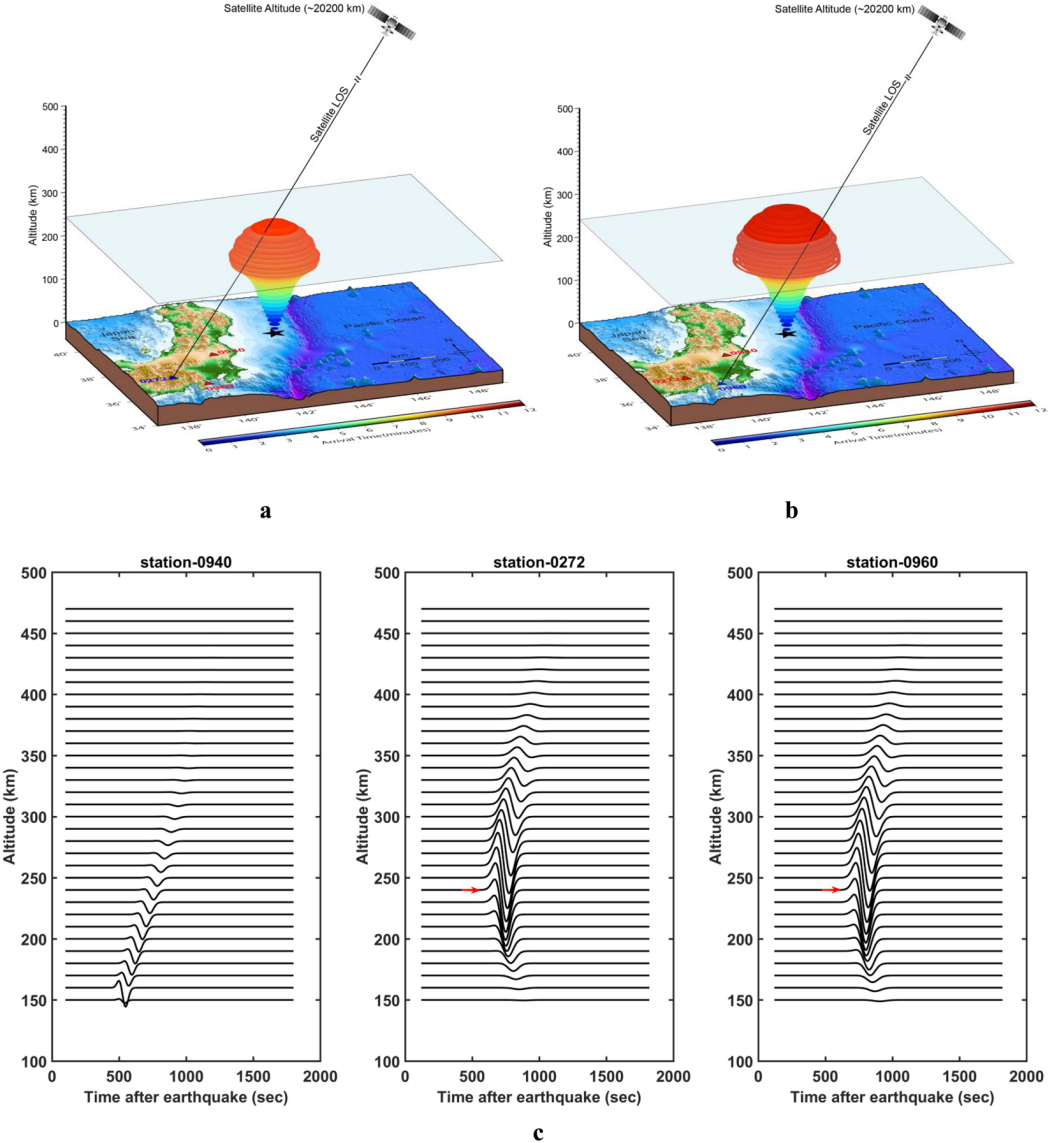
(**a**) Same as Fig. 3 but for GPS station 0272, located ~479 km from the source. The separation between the station (0272)-source azimuthal plane and the station (0272)–satellite azimuthal plane is ~2° at CIP onset at 0272 station. (**b**) Same as Fig. 3 but demonstrates validation of the proposed simple 3D acoustic ray tracing modelling approach for higher separation between the station (0960)-source azimuthal plane and the station (0960)-satellite azimuthal plane (~15°). GPS station 0960 is located ~470 km from the source. (**c**) The altitudinal profiles of synthetic CIP waveforms for PRN 07 for 0940, 0272 and 0960 stations. The synthetic waveforms are from ionospheric altitudes of ~150 km onwards due to limitations in the current 3D spherical modeling software. However, at 0272 and 0960 GPS stations, the synthetic CIP waveforms correlate well to the onset time and associated altitudes derived using the simple 3D acoustic ray tracing model.

We follow the identical steps performed in the case of GPS station 0940 to find the first interaction of the satellite LOS and seismo-acoustic rays for the other GPS stations in this study. Figure 4a presents the CIP detection by PRN 07 at station 0272, which is located ~479 km from the source. The station distance from the maximum uplift source is computed by considering the earth as a sphere. The first interaction between the vertically propagating seismo-acoustic rays and the LOS from station 0272 occurs at ~240 km of altitude ~601 s (~10.01 minutes) after the foreshock event. The onset (Fig. 2c) in CIP time series at station 0272 is ~564 s (~9.4 minutes).

It is pertinent to note that the azimuth of PRN 07 at CIP onset is ~61**°** for station 0940, which is very near to the azimuth of the station(0940)-source line (~63°). In case of station 0272, the station-satellite azimuth is ~58° and the azimuth of the station-source line is ~56.5°. This peculiar satellite geometry for GPS stations 0940 and 0272 of very little separation between the above two mentioned azimuthal planes provide an opportunity to observe the seismic induced acoustic waves over the source. Although the elevation angle of PRN 07 from 0940 is higher (~30.52°) compared to that from 0272 (~27.82°), the corresponding CIP detection is earlier at station 0940. This is due to the fact that 0940 is closer to the epicentre (~251 km). Hence, we suggest for the first time that in addition to the low satellite elevation, satellite azimuth and station distance also play an important role in early CIP detection. For a large azimuthal separation between the station-source and the station-satellite planes at the time of CIP onset, the satellite LOS does not pass through the source area. For these occasions, the LOS intersects the seismo-acoustic rays far away from the source. We support this proposition for the azimuthal separation of ~15° between the station-source and station-satellite planes at the CIP onset at 0960 GPS station during the foreshock and present in the next section.

It is important to note that our 3D acoustic ray tracing modelling approach is based on the space and time evolution of seismo-acoustic rays to explain the early CIP detection. In order to put our simple approach on a firmer footing, we reconstruct the synthetic CIP waveforms on a 3D spherical grid in the presence of a geomagnetic field for a satellite in motion^14,15^ (Fig. 2a–c) and validate the proposed 3D acoustic ray tracing modelling approach in the subsequent section.

### Validation

During the Sanriku-Oki Tohoku foreshock the azimuths of PRN 07 from 0940 and 0272 stations were very near to that of the station-source line during CIP onset at the respective stations. In other words the LOS was almost falling into the azimuthal plane of station-source line. In order to validate our proposed simple 3D modelling approach for azimuthal planes different than that of the station-source line, we apply the same to another station − 0960 (Fig. 4b). It is important to note that the azimuth of the PRN 07 LOS was ~57.7° at the onset of the CIP observed from station 0960, while the station-source azimuth was ~43°. The ODT of CIP onset at 0960 is ~594 s (~9.9 minutes). In Fig. 4b, the first interaction between the PRN 07 LOS and the propagating acoustic ray occurs at an altitude of ~238 km at ~645 s (~10.75 minutes). It is important to note that for station 0272 the interaction between the LOS and the ray occurred at ~240 km and ~601 s (~10.01 minutes), while for station 0960 the interaction occurred at ~238 km at ~645 s.

We suggest that although the PRN 07 LOS for station 0960 was not passing through the source region, the low elevation geometry provided an opportunity to detect the CIP at lower altitude (Fig. 4b). It should be noted that CIP onset detection altitudes at 0272 and 0960 are very similar, but interaction between PRN 07 LOS and acoustic rays occurred after ~44 s at station 0960 than at station 0272. For an altitude difference of ~2 km, the MAT difference would be ~2.5 s as evident from the acoustic wave velocity profile in Fig. S2. In this view, we attribute the difference in MATs of ~44 s to the azimuth of the PRN 07 LOS at 0960 station at the time of the interaction between LOS and acoustic rays. In the final step, we validate the obtained results from the simple 3D ray tracing model (Figs 3 and 4a,b) by comparing it with that of the synthetic CIP waveforms derived using a 3D spherical model which includes a moving satellite geometry in the presence of the geomagnetic field (Figs 2a–c and 4c). The altitudinal profiles of synthetic CIP waveforms for PRN 07 at 0940, 0272 and 0960 GPS stations are presented in Fig. 4c. It is pertinent to note that onset of the CIP at station 0272 is at ~240 km altitude (highlighted with red arrow) and the altitude for the first interaction of the satellite LOS and the acoustic ray in Fig. 4a is also at ~240 km. Similarly, for station 0960 the onset altitude from the synthetic CIP waveform is ~240 km and is ~238 km from the 3D acoustic ray tracing model. Therefore, the CIP waveforms reconstructed by taking into consideration satellite motion and the geomagnetic field support the detection altitude inferred from the proposed 3D simple acoustic ray tracing approach. Additionally, the CIP onsets in integrated synthetic waveforms correlate reasonably well to the observed CIP onsets (Fig. 2a–c).

The difference between the ODT and the first interaction between the PRN 07 LOS for the case studies presented here is within ~51 s. Additionally, the ODT and the onsets of the CIP in the synthetic waveforms for these stations have discrepancies less than ~48 s. We attribute this residual time discrepancy to the model derived temperature and density parameters which are used to estimate the acoustic wave velocity profile (Fig. S2). Apart from this, the horizontal winds also play an important role in changing the acoustic ray travel time^15^. The meridional and zonal winds at the earthquake time are derived using the Horizontal Wind Model (HWM)^22^ and are presented in Fig. S3. We note that the winds were predominantly south-west at the time of the earthquake. The acoustic rays (Figs 3 and 4a,b) and synthetic CIP waveforms (Figs 2a–c, 4c) presented in this study are modelled without taking these winds into account. We assume here that the wind effects become more significant for the increasing difference of station-satellite azimuth plane from that of the source-station and thus for 0960 station the difference between the ODT and the first interaction between the LOS and seismo-acoustic rays is the maximum of ~51 s. It is important to mention that the simple 3D acoustic ray tracing model derives the CIP detection altitude of ~131 km at station 0940 (Fig. 3). However, the synthetic waveforms are from ionospheric altitudes ~150 km onwards due to limitations in the current 3D spherical modeling software.

So far, the satellite geometry is believed to play a major role in controlling the CIP development around the epicentre^4,14^. In the present study we demonstrate through simple 3D acoustic ray tracing modelling that the low elevation satellite geometry and station location with respect to the seismic source explain the observed early CIP manifestation times and help to determine the detection altitudes of CIP onset observed through GPS-TEC.

## Conclusion

We study the co-seismic ionospheric response to the Mw 7.4 Sanriku-Oki Tohoku foreshock occurred on 9 March 2011 and address the specific feature of the early detection of GPS-TEC derived CIP. The azimuthal distribution of CIP amplitudes is in consonance with the uplift deformation pattern over the epicentre and geomagnetic field-acoustic wave coupling at ionospheric altitudes. Using a simple 3D acoustic ray tracing model, we not only explain the early CIP detection during the Sanriku-Oki Tohoku foreshock, but also determine that the detection altitudes of CIP are much lower than the altitude of maximum ionization of the surrounding ionosphere. We validate these results using the synthetic CIP waveforms constructed by taking into consideration a moving satellite geometry and the geomagnetic field. With this, we reveal that GPS based TEC observations provide accurate means to detect the seismo-acoustically induced CIP manifestations in terms of altitude and arrival time, relying only on information about the source location and the ambient atmospheric parameters.

## Methodology

### Crustal deformation pattern during the Mw 7.4 Sanriku-Oki Tohoku foreshock

To obtain the co-seismic ground displacement field over the Japanese Island (black vectors in Fig. 1a), we use GPS data from GEONET. Data were analysed with the GIPSY/OASIS-II Version 6.1.1 software, developed at the Jet Propulsion Laboratory (JPL). We estimated the station coordinates in precise point positioning (PPP) mode^23^. Co-seismic horizontal component offset at the time of earthquake we derived by differencing the mean position 8 days before and 1 day after the foreshock. The observed displacement field of GPS velocity reveals that Japan’s northeast coast moved east-southeast up to ~3 cm during this event. This estimated ground displacement correlates well with previous studies^9^.

To visualize the deformation pattern over the epicentre and rupture area (off-shore), the standard approach of rectangular half-space dislocation modelling of the surface deformation induced by an earthquake is adopted^16^. We estimate the deformation by using the fault parameters on a 80 km × 104 km rectangular plane with a strike of 190° and dip 11° to the west^12^. Our inferred model explains the observed GPS vectors very well (Fig. 1a).

### Slant TEC

Slant TEC (sTEC) represents an integrated ionospheric electron density along the LOS from satellite to receiver. We estimate sTEC using the carrier phase signal observations at GEONET GPS stations^24^. The following formula is used to obtain sTEC from the carrier phases,

1 $$sTEC=\frac{1}{40.3}\times (\frac{{f}_{1}^{2}\times {f}_{2}^{2}}{{f}_{1}^{2}-{f}_{2}^{2}})({L}_{1}\times {\lambda }_{1}-{L}_{2}\times {\lambda }_{2})$$

where *f*_1_ and *f*_2_ are carrier wave frequencies (1.2 GHz and 1.5 GHz, respectively), *λ*_1_ and *λ*_2_ are the corresponding wavelengths, and L_1_ and L_2_ are the carrier phases. Since GPS time is not in accordance with the UTC time, we correct the obtained sTEC time for leap seconds of ~16 s. The frequency filtering of sTEC alters the onset times of CIP which is the main element of this paper, thus we do not apply any frequency filter on sTEC observations.

### 3D acoustic ray tracing model for seismo-acoustic waves

We use the ray tracing method based on the wave refraction phenomenon in varying temperature and density media (i.e. varying velocity media) to estimate the arrival time of propagating seismo-acoustic waves at various atmospheric altitudes^3,4,25^. The maximum crustal uplift, during the Mw 7.4 Sanriku-Oki Tohoku foreshock, is considered as the source for seismo-acoustic wave. The acoustic wave speed mainly depends on the ambient temperature and density through

2 $${\rm{V}}=\sqrt{\frac{\gamma RT}{M}}$$

where $,\,{\rm{\gamma }}$ is specific heat capacity, R is universal gas constant, T is temperature and M is molecular mass density.

In the present case, we obtain the neutral atmospheric temperature and density from the NRLMSISE-00 model^26^ and estimate the seismo-acoustic wave velocity using equation (2). The estimated velocity profile is shown in Fig. S2. We note that the speed of acoustic waves varies significantly with altitudes, which causes the refraction of the waves at each altitude. This refraction ultimately changes the wave propagation direction as the wave propagates upward from the surface of Earth. The arrival times are computed every 1 km of atmospheric altitude. The raypaths of the seismo-acoustic waves are estimated in 3D space.

### Reconstruction of co-seismic ionospheric perturbations

We reconstruct the CIP recorded by PRN 07 using modeling approach proposed by Rolland *et al*.^14^. This model considers an isotropic acoustic point source at the Earth surface, where an initial motion of an N-shape waveform is assumed^27^. The width of the source-time function increases linearly with time propagation (a pulse broadening factor b = 0.04) to take into account the effect of viscous and thermal losses on the phase of the wave (see equation 1 of Rolland *et al*.^14^). The model adopted here enables reconstructing of electron density perturbations on a 3D spherical grid and performs LOS integration for a moving satellite in the presence of a geomagnetic field. The acoustic wave speed is derived using the method described as above. Background electron density and geomagnetic fields are obtained from the IRI-2016^18^ and IGRF models^28^, respectively. The electron density perturbation derived at each altitude is then integrated along the LOS of PRN07 (Fig. 2a–c) to reconstruct the sTEC perturbations, accommodating the satellite motion described by ultra-rapid ephemeris. The altitudinal profiles of CIP waveforms for PRN 07 at 0940, 0272 and 0960 stations are shown Fig. 4c. The temporal resolution of the modelled waveforms is 5 s. Further details of the 3D spherical grid model can be found in Rolland *et al*.^14^ and Dautermann *et al*.^27^.

## Electronic supplementary material

Movie1

Figures S1-S3 and Movie1

## Data Availability

GEONET 1-Hz GPS data used here are available from LR.
