# Author Correction: Revelation of early detection of co-seismic ionospheric perturbations in GPS-TEC from realistic modelling approach: Case study

**DOI:** 10.1038/s41598-018-34742-8

**Published:** 2018-11-01

**Authors:** Dhanya Thomas, Mala S. Bagiya, Poikayil Sukumaran Sunil, Lucie Rolland, Anakuzhikkal Sudarsanan Sunil, T. Dylan Mikesell, Srinivas Nayak, Subrahmanyam Mangalampalli, Durbha Sai Ramesh

**Affiliations:** 1 Indian Institute of Geomagnetism, Navi Mumbai, India; 2 Université Côte d’Azur, OCA, CNRS, IRD, Géoazur, Sophia-Antipolis, Valbonne, France; 3 Environmental Seismology Laboratory, Department of Geosciences, Boise State University, Boise, Idaho USA; 4 Department of Geophysics, Andhra University, Visakhapatnam, India; 5 Department of Marine Geology and Geophysics, Cochin University of Science and Technology, Kochi, India

Correction to: *Scientific Reports* 10.1038/s41598-018-30476-9, published online 14 August 2018

In the original version of this Article, the ORCID IDs for Mala S. Bagiya and Anakuzhikkal Sudarsanan Sunil were omitted.

This has now been corrected in the HTML and PDF versions of the Article.

