# Supplementary material for: Revelation of early detection of co-seismic ionospheric perturbations in GPS-TEC from realistic modelling approach: Case study
Source: Sci Rep. 2018 Aug 14;8:12105. doi: 10.1038/s41598-018-30476-9 (PMC6092341; doi:10.1038/s41598-018-30476-9)
Supplement: Supplementary file 2 — Figures S1-S3 and Movie1 [file 41598_2018_30476_MOESM2_ESM.doc]

**Supplementary Information**

**Revelation of early detection of co-seismic ionospheric perturbations in GPS-TEC from realistic modelling approach: Case study**

Dhanya Thomas^1^, Mala S. Bagiya^1*^, P. S. Sunil^1^, Lucie Rolland^2^, A. S. Sunil^1^, T.Dylan Mikesell^3^, Srinivas Nayak^1^, M. Subrahmanyam^4^, D. S. Ramesh^1^

^1^Indian Institute of Geomagnetism, Navi Mumbai, India

^2^Université Côte d’Azur, OCA, CNRS, IRD, Géoazur, Sophia-Antipolis, Valbonne, France

^3^Environmental Seismology Laboratory, Department of Geosciences, Boise State University, Boise, Idaho, USA.

^4^Department of Geophysics, Andhra University, Visakhapatnam, India

*Corresponding Author: [bagiyamala@gmail.com](mailto:bagiyamala@gmail.com)

**Supplementary**

**Figures S1, S2, S3 and Movie 1.**

**
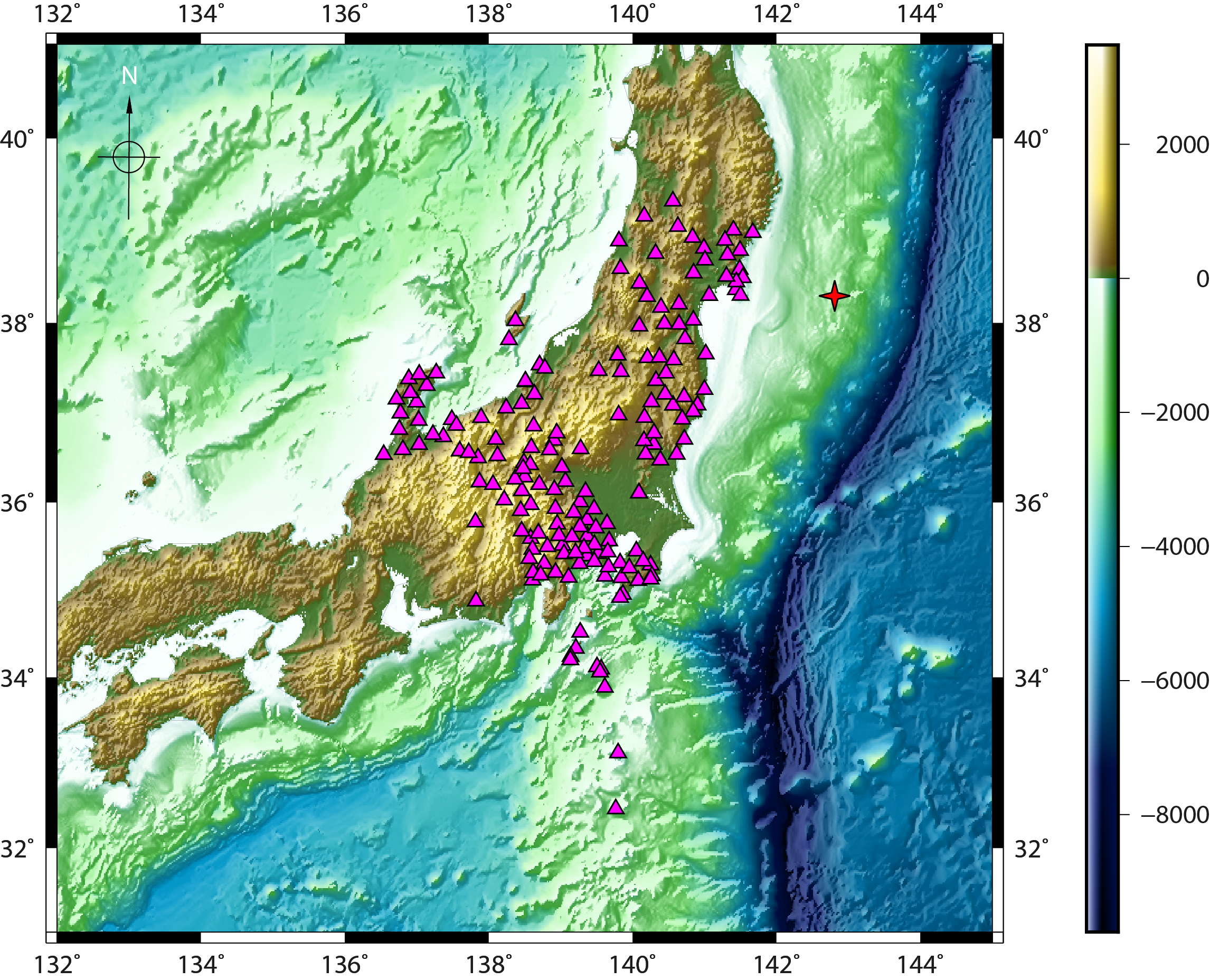
**

**Figure S1:** Locations of the 173 GEONET stations used in this study to observe the co-seismic ionospheric response during the Mw7.4 Sanriku-Oki Tohoku foreshock. The location of the epicentre is indicated by a red star. The map is generated using the Generic Mapping Tools^29^.

**
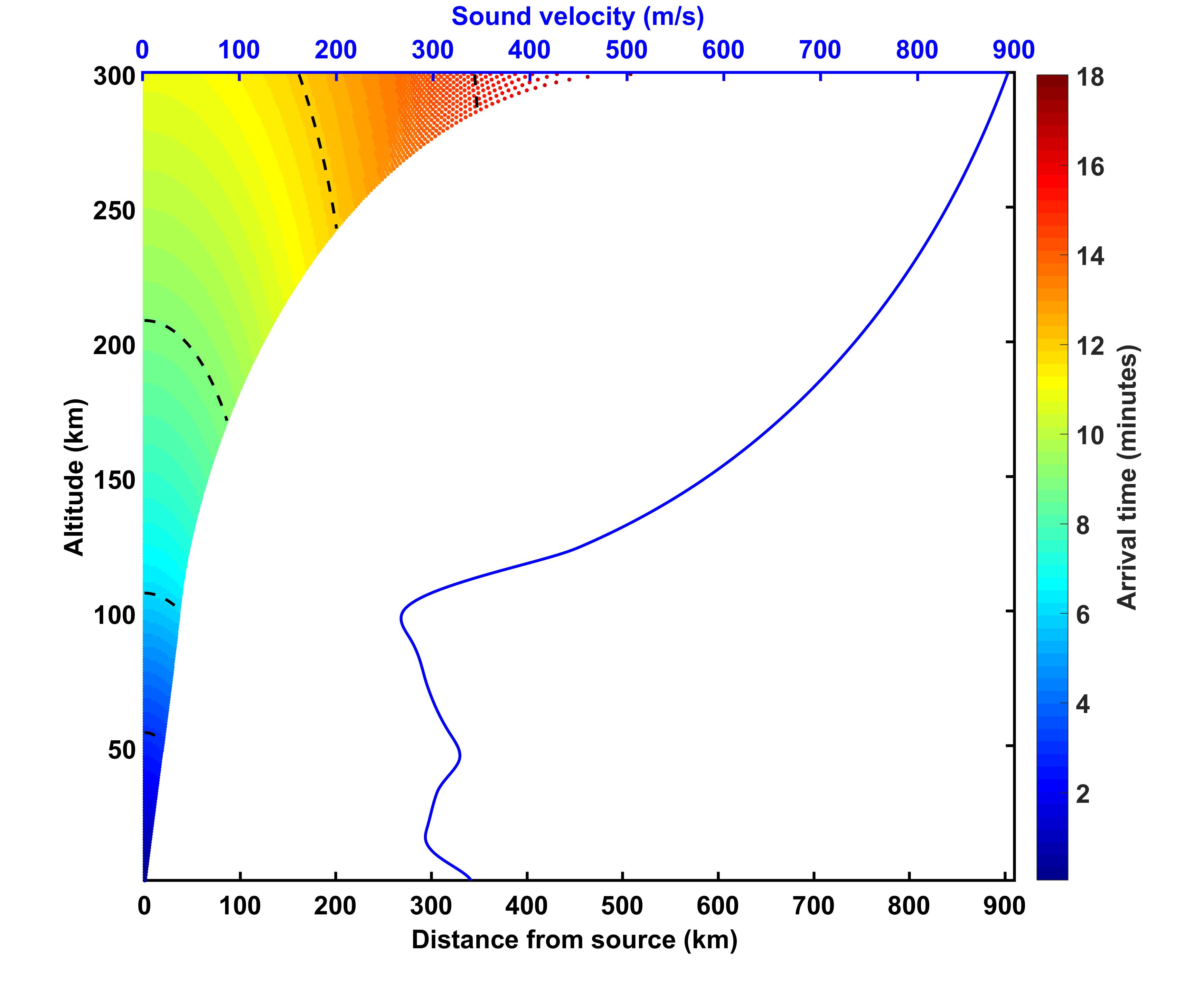
**

**Figure S2:** Vertical cross section of the altitudinal evolution of seismo-acoustic rays along with the acoustic wave velocity profile used to trace the rays at various atmospheric altitudes.

**
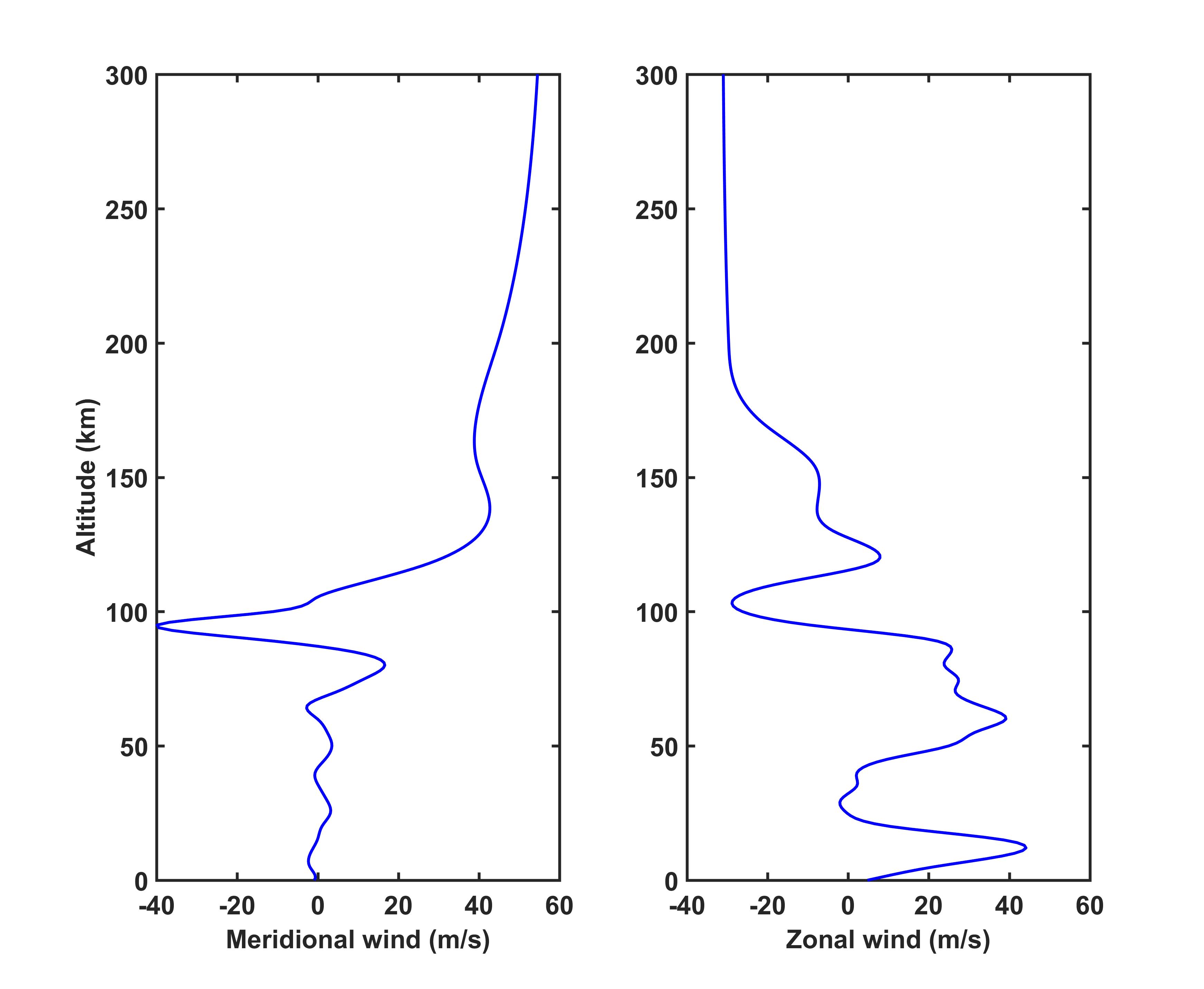
**

**Figure S3:** Meridional and Zonal winds, respectively, extracted from the HWM model at the time of earthquake onset.

**Movie1:** Temporal evolution of CIP after 400s from the earthquake onset time (02:45:20 UTC) as observed by PRNs 07, 08 and 10 from GEONET stations (Figure S1a). The evolution is presented at every 25s. The background color is the modeled arrival time at the IPP altitude of ~274 km computed using the 3D acoustic ray tracing method and considering the maximum uplift as the source. Black five point star indicates the location of maximum uplift estimated in this study and the red four point star indicates the epicentre during the foreshock obtained from the USGS earthquake catalogue (https://earthquake.usgs.gov).
